# Musculoskeletal pain and exercise—challenging existing paradigms and introducing new

**DOI:** 10.1136/bjsports-2017-098983

**Published:** 2018-06-20

**Authors:** Benjamin E Smith, Paul Hendrick, Marcus Bateman, Sinead Holden, Chris Littlewood, Toby O Smith, Pip Logan

**Affiliations:** 1 Physiotherapy Department (Level 3), Derby Hospitals NHS Foundation Trust, London Road Community Hospital, Derby, UK; 2 Division of Rehabilitation and Ageing, School of Medicine, University of Nottingham, Nottingham, UK; 3 Division of Physiotherapy and Rehabilitation Sciences, School of Health Sciences, University of Nottingham, Nottingham University Hospitals, Nottingham, UK; 4 Research Unit for General Practice in Aalborg, Department of Clinical Medicine, Aalborg University, Aalborg, Denmark; 5 SMI, Department of Health Science and Technology, Aalborg University, Aalborg, Denmark; 6 Arthritis Research UK Primary Care Centre, Research Institute for Primary Care & Health Sciences and Keele Clinical Trials Unit, Keele University, Staffordshire, UK; 7 Nuffield Department of Orthopaedics, Rheumatology and Musculoskeletal Sciences, University of Oxford, Oxford, UK

**Keywords:** chronic, exercise, exercise rehabilitation, review

## Introduction

Chronic musculoskeletal pain remains a huge challenge for clinicians and researchers. Exercise interventions are the cornerstone of management for musculoskeletal pain conditions,^1^ with the benefits being well-established.^1 2^ Exact mechanisms underpinning this effect on musculoskeletal pain are currently unclear.^3^ Little is known on the optimal dose and type of exercise, with therapists’ and patients’ behaviour and beliefs around pain during exercise often overlooked in exercise prescription. Exercise-based treatments may be promising, but effect sizes remain small to modest with large variability in exercise prescriptions.

The need for pain to be avoided or alleviated as much as possible has been challenged, with a paradigm shift from traditional biomedical models of pain towards a biopsychosocial model of pain, which is particularly relevant in the context of performing therapeutic exercise.^4^ Indeed, a recent systematic review and meta-analysis of painful exercises versus pain free exercises for chronic musculoskeletal pain that included seven randomised controlled trials found that protocols allowing painful exercises offered a small, but statistically significant, benefit over pain-free exercises in the short-term.^4^ The improvements in patient-reported pain were achieved with a range of contextual factors, such as varying degrees of pain experienced (ranging from pain being allowed to advised, with/without recommended pain scale) and recovery time (ranging from pain subsiding immediately to within 24 hours). Specifically, we define painful exercises when: exercises are prescribed with instructions for patients to experience pain or where patients are told that it is acceptable and safe to experience pain.

Understanding the potential mechanisms behind the effects of therapeutic exercise, in the context of factors associated with chronic musculoskeletal pain, is key to optimising current exercise prescriptions for managing musculoskeletal pain. The aim of the review is to provide an understanding on the potential mechanisms behind exercise and to build on this into discussing the additional theoretical mechanisms of painful exercises.

This narrative review provides an overview of the current understanding of:

- Musculoskeletal pain in relation to central and peripheral pain mechanisms, the immune system and affective aspects of pain, see box 1 for summary. This review focuses on these three mechanisms as these systems may respond differently to painful stimulus, compared with a non-painful stimulus^5–8^;
- Then, the proposed mechanisms behind the potentially additional beneficial effect of allowing painful exercises over pain free exercises for individuals with chronic musculoskeletal pain.

## Brief background into our current understanding of chronic pain

### Mechanisms of central and peripheral sensitisation

Central sensitisation typically describes an increased responsiveness of nociceptive neurons in the central nervous system (CNS) to normal input. With central sensitisation, there are changes in the properties and function of neurons in the CNS, with an increase in pain response relative to the presence and intensity of noxious peripheral stimuli.^9 10^

In humans and clinical studies, we can measure surrogates which are thought to be reflective of central sensitisation and cover many different underpinning mechanisms.^9^ Central sensitisation can be seen as an umbrella term,^9^ the main characteristics of which are:

- hyperalgesia;
- allodynia;
- temporal summation of pain (TSP) and
- diffuse noxious inhibitory control (DNIC).^9–12^

Hyperalgesia is an increased pain response to normally painful stimuli and may be as a result of increased peripheral or central pain sensitivity.^13^ If someone were to experience a pin prick to their knee, they may score the pain one out of 10, for example. However, if they were suffering with chronic knee pain, with hyperalgesia, the same pin prick stimuli would result in a more painful response and a higher pain score being reported.

Allodynia, by contrast, is a pain response to a stimulus that is not normally painful.^10 14^ An example of allodynia is the person who is suffering from chronic low back pain who complains of pain when they are hugged.

TSP is a progressive increase in pain perception in the response to repeated stimuli of the same intensity and thought to represent central pain facilitation occurring at the dorsal horn neurons when integrating the incoming nociception.^11^ A variety of stimuli can be used to assess temporal summation in humans, including heat, cold, pressure and electrical. For example, a patient with chronic knee pain performing knee exercises may complain of increasing levels of pain the more repetitions of the same exercise they perform, which could be attributed to TSP.

Another commonly assessed pain mechanism in musculoskeletal pain research is the DNIC paradigm.^12^ It describes a descending endogenous pain modulation system encompassing an array of overlapping mechanisms from the CNS that may modulate and inhibit pain.^15^ The two main mechanisms are the activation of descending nociceptive inhibitory mechanisms^16^ and the release of endogenous opioids.^17^ DNIC can be assessed in humans through the conditioned pain modulation (CPM) response (also known as ‘pain inhibits pain’). During CPM, the descending pain inhibitory responses are challenged during a painful conditioning stimulus. This is used as a proxy of the overall effectiveness of the endogenous analgesic system, likely occurring through both the opioid and non-opioid pathways. An example of CPM in action is when one might report lower pain scores for a primary complaint, say low back pain, in the presence of a secondary painful stimulus, for instance placing the hand in ice cold water.

### The role of the immune system

It is thought the immune system plays an important role in chronic pain states, including the development of long-term hyperalgesia and allodynia.^18–20^

The innate immune response of inflammation is activated by various processes, including exposure to microbial cell wall fragments, toxins, irritant chemicals and autoimmune reactions.^21^ Typically, these are detected by a family of pattern-recognition receptors called toll-like receptors (TLRs) that regulate the CNS’s innate immune response.^19^ TLRs are predominantly made up of glial cells and sense the presence of damage or danger originating both endogenously and exogenously, translating this into central immune signals that can be interpreted by the CNS.^18 20^

A process by which the immune system may influence hyperalgesia and allodynia is through alterations of glial cells from a normal immune function to being capable of acting on dorsal horn neurons as a nociceptor.^9^ Some studies report increased glial activity with individuals with chronic pain.^18^ The mechanisms by which glial cell activation leads to synaptic plasticity are not fully understood, but this pathological pain state is thought to correlate with central sensitisation, with a large overlap of contributing mechanisms.^18^

### Affective aspects of pain

Identification of pain-related fear and negative emotional states, such as kinesiophobia, catastrophising, low self-efficacy, anxiety and depression, are becoming increasingly recognised in some musculoskeletal disorders.^22 23^ Research has shown that these psychological factors might affect the function and quality of life in patients with pain and can modulate the individuals’ pain experience and therefore may play a role in the development and/or maintenance of chronic pain states.^24–30^ A systematic review of self-management interventions for chronic musculoskeletal pain (16 studies; n=4047) found self-efficacy and depression were the strongest prognostic factors (irrespective of the intervention).^31^ Reducing pain catastrophising and increasing physical activity were the strongest mediating factors, that is, factors which may explain how different treatments may work.^31^

Pain can negatively affect physical activity and mental thought processes and requires cognitive resources.^32 33^ It has been proposed that pain-related fear amplifies the experience of pain; indeed there is strong evidence that pain is experienced more strongly when there is a greater focus of attention on it.^34–38^ A person with pain-related fear may have a greater amount of attention bias, by which it means they pay the pain greater attention, with greater emotional meaning attached to it.^25^ The mechanisms by which pain-related fear is thought to influence central sensitisation are: (1) increasing nociceptive transmission via spinal gate mechanism^39^; (2) via modulation of the descending pathways^39^ and (3) temporal summation, where increasing magnitude of spinal dorsal horn neurons activation increases glutamine sensitivity, thus producing a pain response disproportionate to the stimulus experienced.^9 25^ Indeed, evidence from neuroimaging has demonstrated the role of the amygdala and pain-related fear, and its potential over activity, as a facilitator of chronic pain and central sensitisation.^40–42^

Box 1 **Summary—Pain Science 2018 in a nutshell.**

Traditional pain models that describe tissue pathology as a source of nocioceptive input directly linked with pain expression are insufficient for assessing and treating musculoskeletal pain.^75^ Other models reconceptualise pain and put forward concepts that are based on the premise that pain does not always provide a measure of the state of tissue pathology. Instead, pain is modulated by many factors, and the relationship between pain and tissue becomes less predictable the longer pain persists.^30^ Altered central processing of pain has been shown to be present in many pain conditions,^76-83^ with the immune system playing a role in the development and maintenance of pain sensitisation.^18–20^ Furthermore, unhelpful thoughts of patients and clinicians towards pain, including belief that pain will not get better and that movement will cause further tissue damage and worsening of the pain, are also important issues to remain mindful of.^22 23^

## How might allowing painful exercise mitigate pain? Three mechanisms that arise from recent neuroscience discoveries

Traditional explanations by which exercise improves pain and disability in chronic musculoskeletal pain rely on its effect on biomechanics and corresponding changes in loading of the musculoskeletal system.^2^ This model of clinical reasoning, whereby pain improves as a result of biomechanics, fails to take into account the full biopsychosocial spectrum of factors. This may be the reason why there is a lack of evidence supporting any specific exercise intervention. It may be that factors common to all exercises have the greatest mediating effect on pain and disability. The following section will discuss the mechanisms associated with exercise and central pain processes, the immune system and affective aspects of pain, including a theoretical rationale for the potential additional benefit of allowing painful therapeutic exercise, over and above pain free exercises alone.

### Affective aspects of pain—reconceptualisation of pain-related fear

Some patients report fear of doing further tissue damage if an activity or exercise is painful.^43–45^ A major consideration of the beneficial effects of painful exercise is the potential associated learning involved. Painful exercises have the potential to help reconceptualise pain-related fear, that is, patients may be challenged to think differently about pain and tissue damage, and allowing painful exercises offers an opportunity for patients to reintroduce movement that were previously perceived as a threat. The amygdala is often referred to as the fear centre of the brain^5^ and plays a key role in shaping our response to fear, particularly our response to pain-related memories and fear.^5^ The cingulate cortex also plays a role in our response,^42^ with both areas of the brain communicating directly via the descending nociceptive inhibitory system.^24 46 47^ In chronic pain states, the brain acquires long-term maladaptive pain memories that associate tissue stress and load with danger and threat,^48^ for example, bending forwards in individuals with low back pain, raising the arm or lifting objects with shoulder pain or squatting type movements with individuals with knee pain.

Contemporary thinking in relation to movement adaptation and pain argue that activity avoidance precedes the development of pain, with pain causing the behavioural changes.^49^ However, research has demonstrated that even mental preparation for such movements and activities can trigger the fear-memory centre of the brain, thought to be an overactive threat protective mechanism, triggering pain, even in the clear absence of nociception.^50^ This is an important finding, as it links with other work that has demonstrated that an individuals’ beliefs and attitude to pain, and what constitutes ‘threatening’ pain or not, leads to altered movement behaviour in those that perceive a stimulus as threatening.^51^

By allowing painful exercises, with appropriate ‘safety-cues’, new inhibitory associations may be made; these new inhibitory associations theoretically may compete with the original conditioned response, so that it becomes suppressed.^52^ Safety-cues may include statements such as: ‘your shoulder is painful because it has become deconditioned and not used to movement. We need to exercise your shoulder, so it will become strong and conditioned to enable you to do what you need to do’. Research supporting this concept has come from animal studies^53 54^ that have reported involvement of the medial prefrontal cortex (mPFC) in the learning of new inhibitory associations, which has direct projections onto the amygdala.^52^ For instance, the mPFC might have a role in the storing of long-term extinction memories that block and suppress the amygdala. Human studies on military personnel with and without a clinical diagnosis of posttraumatic stress disorder (PTSD) have confirmed this inverse relation between activity in the mPFC and amygdala.^55^ Patients with PTSD had decreased activation of the mPFC, with correlated increased activation of the amygdala.^55^ Clinically, this is an important point, since it highlights that despite a positive response to therapy, pain-related fear may never truly been eliminated. It may, given certain conditions, for example during an acute flare up, resurface.

It is thought that allowing painful therapeutic exercises could reduce the threat perception, and thus the activity of the amygdala and somatosensory cortex,^56^ with positive modulation of the nociceptive inhibitory systems. An example of this in practice would be providing safety-cues to a patient who is fearful of lifting a painful shoulder they have been resting for long periods.

Self-efficacy, one’s ability to cope, another psychosocial factor associated with pain-related fear, may also be used to explain fear reduction. As previously discussed, self-efficacy is a key prognostic factor for success of self-management interventions for musculoskeletal pain.^31^ The potential mechanisms behind the effect of painful exercises are thought to be that painful exercises may alter both the response-outcome and efficacy expectation, both components of self-efficacy.^57^ Within the context of the theory presented, the hierarchy construction of painful exercises, from easier to more difficult/higher load, could improve one’s response-outcome expectation, where the patient begins to expect that they can tolerate harder exercises, without triggering the previous experience of pain-related fear and pain flare-ups.^58^

### Central pain processes

It has been recognised that an acute bout of exercise can result in analgesia and this phenomenon is termed exercise-induced hypoalgesia (EIH) and is one form of endogenous pain modulatory processes.^59^ It is thought that EIH is dependent on multiple analgesic mechanisms that contribute to changes in pain sensitivity.^60^ Evidence for the analgesic effect of exercise comes from experimental studies that attenuate pain sensitivity, as measured by pressure pain thresholds and temporal summation.^11 60 61^ A number of different exercise interventions have been investigated, including cardiovascular exercise (running and cycling) and resistance exercise, including isometric and dynamic resistance.^59^ It is thought the endogenous opioid system is triggered by exercise-induced activation of arterial baroreceptors following increases in heart rate and blood pressure, with an associated dose response.^3 62 63^ Exercise can trigger the release of β-endorphins from the pituitary and hypothalamus, in turn activating µ-opioid receptors peripherally and centrally, triggering the endogenous opioid system.^64^ The hypothalamus projects onto the periaqueductal grey (PAG) resulting in further endogenous analgesic effects via the descending nociceptive inhibitory mechanisms.^3^ A recent systematic review concluded that painful exercises typically have higher loads and dose of exercise^4^ and a theoretical reason painful exercises may have a greater affect than pain free exercises could be a greater EIH.

Another theoretical reason painful exercises may work to reduce pain is through the CPM response. As previously explained, during CPM the descending pain inhibitory responses are challenged during a painful conditioning stimulus.^65^ Several studies have demonstrated that pain-related fear negatively disrupts the endogenous pain inhibitory systems via the process of CPM, for example, higher levels of catastrophising during experimental studies was strongly associated with lower activation of the DNIC and higher pain ratings.^6^ The network of subcortical and cortical structures associated with DNIC and CPM include the amygdala.^66^ Painful exercises could provide the painful conditioning stimulus needed to trigger the CPM response, within the context of reducing pain-related fear (as discussed in the previous section) and activity of the amygdala, which may provide a mechanistic rationale for improvements in pain and function.

### The immune function and pain-related fear

As discussed previously, the immune system may play a role in chronic pain states, and the development of long-term hyperalgesia and allodynia.^18–20^ This section now returns to this topic, in relation to exercise and, specifically, questioning the belief that exercises must be pain-free.

It is well understood that regular general exercise reduces the risk of developing age-related illnesses, such as heart disease and type 2 diabetes.^67^ However, regular general exercise also reduces susceptibility to viral and bacterial infections, suggesting that there are mechanisms at play that improves the overall immune function.^68 69^

Looking specifically at allowing painful exercises, it is known that the amygdala projects onto areas of the brain that play key roles in the sympathetic response to threat, such as the locus coeruleus and pons,^70^ with inflammation being directly activated by the sympathetic nervous system response.^71 72^ For example, two functional MRI studies looking at brain and immune function during experimental periods of induced psychological stress reported increased activity of the amygdala, with subsequent increases of inflammatory markers.^7 8^ Therefore, allowing painful exercises, set within a framework of reducing fear-avoidance, with reconceptualisation of pain-related fear, could reduce the threat perception and thus the activity of the amygdala and somatosensory cortex. The result of which could be positive modulation of the sympathetic nervous system over and above the usual effect of physical activity, and a greater reduction in the cascade of the physiological immune response and the inflammatory system.

Evidence for this comes from studies looking at the sympathetic nervous system’s response to pain-related fear and movement or exercise. For example, during painful movements, patients with persistent pain showed more activation of the right insular cortex, thought to have direct interactions with the sympathetic nervous system, than pain-free controls.^73 74^ Similarly patients with chronic arm pain demonstrated increased swelling, in response to motor imagery, without any actual movements, which was related to fear of pain and catastrophising,^74^ demonstrating that these psychosocial factors may modulate the relationship between the motor and sympathetic system.^74^

## Limitations

This narrative, non-systematic, review has described concepts supported by preliminary data. Many of the mechanisms are similar for both painful and pain free exercises and current evidence shows only modest difference in efficacy.^4^

## Summary

**Figure 1:**
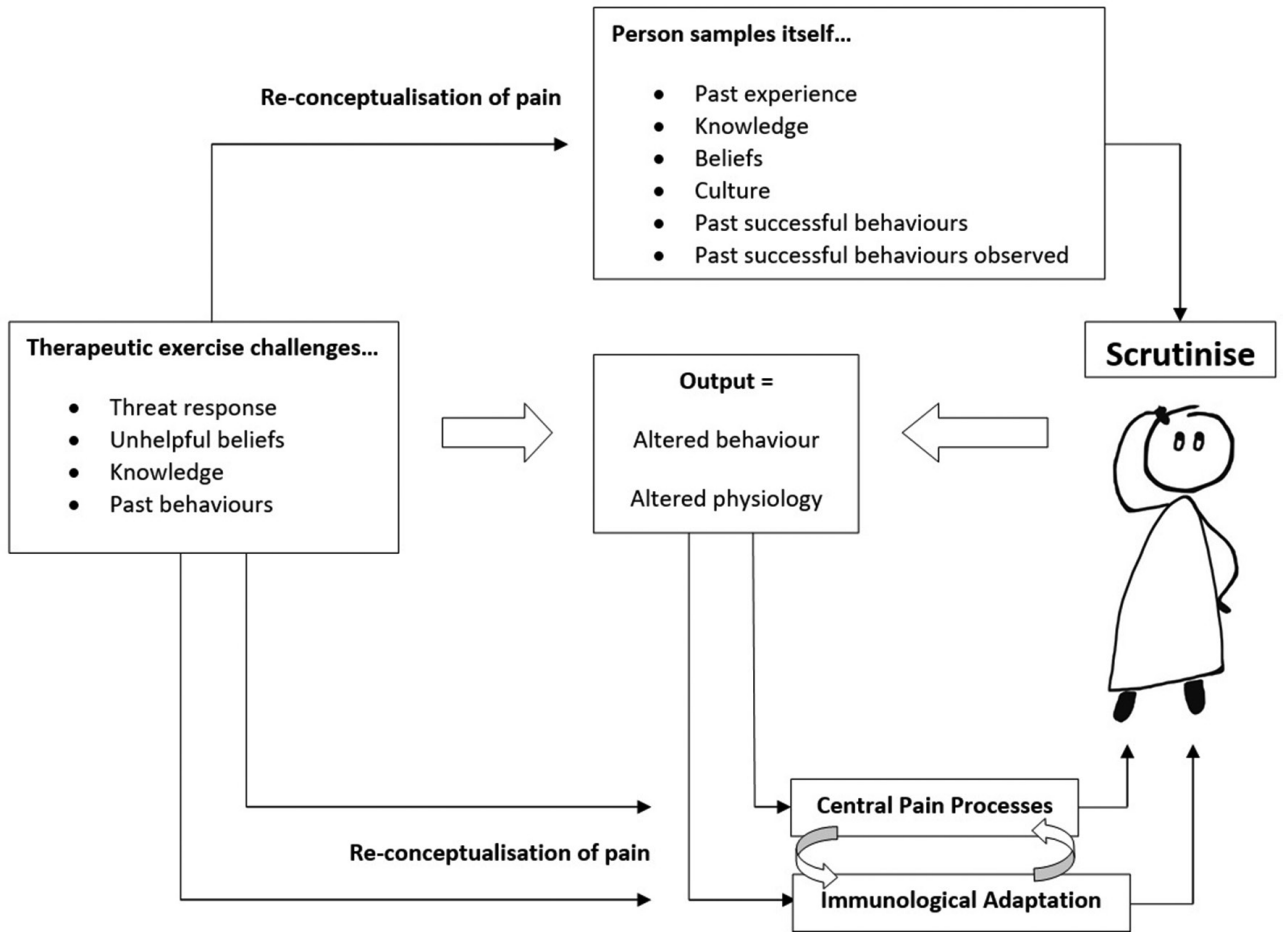
The role of exercises in the management of chronic musculoskeletal pain. Therapeutic exercise challenges the threat response to pain. Central pain processes, the immune system and affective aspects of pain may respond differently when pain is conceptualised as non-threatening. Adapted from Physiotherapy, 84(1), Gifford, Louis., ‘Pain, the tissues and the nervous system: a conceptual model’, 27–36, Copyright (1998), with permission from Elsevier.

Central pain processes, the immune system and affective aspects of pain appear to respond to exercise in a positive way. There might be some additional advantages when the exercise is painful, over and above pain-free. These overlapping mechanisms may mitigate and moderate musculoskeletal pain, and through the delivery of exercises that reconceptualise pain as safe and non-threatening, facilitated by appropriate clinical support and education (figure 1). Allowing painful exercises may result in greater loads/volume of exercise, but does challenge traditional prescription based solely on strength and conditioning principles with a tissue-focussed approach.

## Conclusion and implications

This review has presented a contemporary understanding of musculoskeletal pain towards a potential rationale for the mechanisms behind any additional benefit of allowing painful exercises, over pain-free exercises, in the management of musculoskeletal disease. This additional mechanistic consideration could be used to help clinicians in the prescription of therapeutic exercise (table 1) and for researchers to advance knowledge for such a globally problematic condition.

**Table 1:** How to reconceptualise pain-related fear through exercise—practical solutions

Treatment goal	Example
Understand what the patient understands	Why do you think you have pain?
Challenge unhelpful beliefs	Is it safe for you to exercise? Why? Discuss with the patient. Prescribe exercises or movements that were previously avoided/or painful. New inhibitory associations may be made with painful exercises.
Enhance self-efficacy	Are you confident of completing this exercise? What do you think will happen? Discuss with the patient. The hierarchy construction of painful exercises, from easier to more difficult may improve self-efficacy.
Provide safety-cues	Your knee is painful because it has become deconditioned and not used to movement. Pain is not a sign of tissue damage. We need to exercise your knee, so it will become strong and conditioned to enable you to do what you need to do.
Provide advice on suitable levels of pain	If you’re coping with the level of pain, then continue with the exercise. If the pain is more than you find acceptable or flares up longer than 24 hours after the exercise, then decrease the amount of exercise until you’re coping with it again.
Provide advice on exercise modification	It is important to adjust the exercises dependent on your symptoms. This may mean increasing the number of repetitions that you do or the amount of resistance that you use as it becomes easier; or decreasing if it gets too painful. Try not to avoid doing the exercises altogether as complete rest is unlikely to solve the problem. Instead reduce the exercises to a level that is acceptable.

**What are the new findings?**

- Central and peripheral pain mechanisms, the immune system and affective aspects of pain appear to respond differently when pain is allowed during exercise.
- Pain during therapeutic exercise for chronic musculoskeletal pain need not be a barrier to successful outcomes.
- There is a potential rationale and mechanisms behind the additional benefit of allowing painful exercises, over pain-free exercises, in the management of chronic musculoskeletal pain.
